# HETEROGENEOUS METABOLOMIC AGING ACROSS THE SAME AGE AND PEDICTION OF HEALTH OUTCOME

**DOI:** 10.1093/geroni/igae098.3996

**Published:** 2024-12-31

**Authors:** Xueqing Jia, Jiayao Fan, Xucheng Wu, Joris Deelen, Dan Zhou, Zuyun Liu

**Affiliations:** Zhejiang University School of Medicine, Hangzhou, Zhejiang, China (People’s Republic); Zhejiang University School of Medicine, Hangzhou, Zhejiang, China (People’s Republic); Zhejiang University School of Medicine, Hangzhou, Zhejiang, China (People’s Republic); University of Cologne, Cologne, Nordrhein-Westfalen, Germany; Zhejiang University School of Medicine, Hangzhou, Zhejiang, China (People’s Republic); Zhejiang University School of Medicine, Hangzhou, Zhejiang, China (People’s Republic)

## Abstract

Existing metabolomic clocks exhibit deficiencies in capturing the heterogeneous aging rates among individuals with the same chronological age. Yet, the modifiable and non-modifiable factors in metabolomic aging have not been systematically studied. Here, a new aging measure—MetaboAgeMort—was generated and validated using metabolomic profiles from 239,291 UK Biobank participants for 10-year all-cause mortality prediction. The MetaboAgeMort showed significant associations with all-cause mortality, cause-specific mortality, and diverse incident diseases. Adding MetaboAgeMort to a conventional risk factors model improved the predictive ability of 10-year mortality. A total of 99 modifiable factors across seven categories were identified for MetaboAgeMort. Among these, 16 factors representing pulmonary function, body composition, socioeconomic status, dietary quality, smoking status, alcohol intake, and disease status showed quantitatively stronger associations. The genetic analyses revealed 99 genomic risk loci and 271 genes associated with MetaboAgeMort. The tissue-enrichment analysis showed significant enrichment in liver. This study illuminates heterogeneous metabolomic aging across the same age, providing avenues for identifying high-risk individuals, developing anti-aging therapies, and personalizing interventions, thus promoting healthy aging and longevity.

